# Energy and caloric restriction, and fasting and cancer: a narrative review

**DOI:** 10.1007/s00520-020-05879-y

**Published:** 2020-11-14

**Authors:** Ezzeldin M. Ibrahim, Meteb H. Al-Foheidi, Mubarak M. Al-Mansour

**Affiliations:** 1 Oncology Center, International Medical Center, Jeddah, Kingdom of Saudi Arabia; 2 Princess Noorah Oncology Center, King Abdulaziz Medical City, Ministry of National Guard Health Affairs-Western Region (MNGHA-WR), Jeddah, Kingdom of Saudi Arabia; 3 College of Medicine, King Saud Bin Abdulaziz University for Health Sciences, Jeddah, Kingdom of Saudi Arabia; 4 Adult Medical Oncology, Princess Noorah Oncology Center, King Abdulaziz Medical City, Ministry of National Guard Health Affairs-Western Region, PO Box 9515, Jeddah, 21423 Kingdom of Saudi Arabia

**Keywords:** Energy restriction, Caloric restriction, Fasting, Cancer

## Abstract

Dietary interventions have a significant impact on body metabolism. The sensitivity of cancer cells to nutrient and energy deficiency is an evolving characteristic of cancer biology. Preclinical studies provided robust evidence that energy and caloric restrictions could hinder both cancer growth and progression, besides enhancing the efficacy of chemotherapy and radiation therapy. Moreover, several, albeit low-powered, clinical trials have demonstrated clinical benefits in cancer patients. Future research will inform and firmly establish the potential efficacy and safety of these dietary interventions. Here, we review the current evidence and ongoing research investigating the relationship between various dietary restriction approaches and cancer outcomes.

## Introduction

Diet-related obesity is a known risk factor for numerous aging-related diseases and several types of cancers, including breast cancer, pancreatic cancer, colorectal cancer, hepatic cancer, and prostate cancer [1]. In cancer patients, obesity is associated with an increased risk for disease recurrence and higher disease-specific or overall mortality [2].

Preclinical studies have shown that caloric restriction (CR) can protect animals against the toxic effects of chemotherapy, while also enhancing the efficacy of various chemotherapeutic agents [3].

Thus, this review will examine the current knowledge concerning the mechanisms that explain normal and cancer cells’ responses to CR. Moreover, it summarizes the available preclinical and clinical data that addressed the impact of CR on cancer incidence, and its potential effects on enhancing efficacy and reducing the toxicity of chemotherapy and radiotherapy.

## Review

### Autophagy

To understand the relationship between energy restriction (ER) and cancer biology, a thorough insight of the autophagy is required. Autophagy is a catabolic process essential to support cellular homeostasis and survival in response to both physiologic and pathologic stimuli by involving degradation and recycling of cells’ intracellular endogenous and exogenous components through the lysosomal machinery [4, 5]. This mechanism aids cell survival by providing primary materials including amino acids, fatty acids, nucleosides/nucleotides, sugars, and nucleosides/nucleotides during the state of cellular deprivation [6].

Autophagy operates at basal levels, and it can be induced in response to chemotherapeutic treatment and various cellular conditions including hypoxia, DNA damage, and nutrient depletion [7]. In the past years, the dynamic role of autophagy has gained increasing attention as it could be a therapeutic target for several diseases, including cancer [8].

The preponderance of evidence indicated that autophagy suppresses tumorigenesis and inhibits cancer cell adaptation, proliferation, survival, and invasion or metastasis [9].

Several studies using animal models suggested that the tumor-suppressive mechanism by autophagy was associated with cellular responses from different types of stress, such as oxidative stress, DNA damage, inflammation, and dysfunctional elements. While there is no evidence that autophagy contributes to cancer development, it may have a vital role in tumor survival and progression [10].

Mutations in the tumor suppressor gene p53 have been frequently observed in a wide range of human cancers, which also supports autophagy initiation to recycle intracellular components that subsequently promote tumor growth [11]. Further studies have shown that the upregulation of autophagy is an important protection against chemotherapeutic agents, which is associated with treatment resistance [12, 13].

Autophagy modulation may be considered a new therapeutic strategy in treating patients with cancer [12–15]. The combination of autophagy inhibitors such as bafilomycin A1, chloroquine, or 3-methyladenine with standard agents has shown increased efficacy in treating tumor cells [16–18]. Notably, inhibition of autophagy demonstrated an increase response to radiotherapy in ovarian [19] and esophageal [20] cancer, likely due to enhanced oxidative stress and DNA damage during short-term fasting on cancer cells. Although results from clinical trials have not found an association between autophagy inhibition and anticancer therapy, there are several approved agents known to modulate autophagy [21].

### Cancer cells

Cancer cells demonstrate an increase in anabolic reactions that give rise to the so-called Warburg effect. Tumor cells consume large amounts of glucose even in conditions in which oxidative phosphorylation can proceed unrestrictedly, they are also able to take up large amounts of amino acids [22], and they are avid consumers of lipids [23]. Studies with the human subjects found that long-term caloric restriction (CR) have significant reductions in metabolic and hormonal factors that are likely to be associated with the risk of developing cancer [24, 25].

### Energy restriction (ER), caloric restriction (CR), intermittent energy restriction (IER), and fasting and cancer

There are two most common types of energy restriction (ER), the caloric restriction (CR) or continuous energy restriction (CER) and the intermittent energy restriction (IER). The IER encompasses a variety of fasting patterns, including intermittent fasting, periodic fasting, alternate-day fasting, fasting-mimicking diet (FMD), or time-restricted feeding [26]. Fasting is defined as complete food deprivation except to drink water, with intervals of regular food intake. CR is distinct from fasting in which average daily caloric intake reduces by 20–40% without malnutrition or deprivation of essential nutrients [27].

In response to ER, metabolic changes induce health-promoting effects, including increased insulin sensitivity and decreased blood glucose, growth factor signaling, inflammation, angiogenesis, and protection against oxidative stress [28–30]. Exposure to an ER diet results in reduced systemic glucose and growth factors such as insulin-like growth factor [31], and the latter is known to play a significant role in the development and progression of tumors through the activation of two major signaling cascades, namely Ras/MAPK and PI3K/AKT [32]. CR also induces activation of AMP-activated protein that results in increased apoptosis [33]. Fasting has also been shown to cause an anti-Warburg effect and promote apoptosis in vitro cancer models [34].

The National Institute of Ageing Studies showed that the incidence of cancers in rhesus monkeys fed a CR diet is reduced compared to that in animals fed a control diet [35]. Recent in vitro and in vivo studies have shown that ER significantly enhanced the efficacy of several chemotherapeutic agents [34, 36–38]. It has been postulated that fasting may improve anticancer therapies’ effectiveness in part by controlling the circadian rhythm [39]. In several animal models, intermittent fasting combined with chemotherapy show enhanced suppression of tumor growth and improved overall survival [3]. In xenograft malignancies in mice, tumor growth was slower in response to chemotherapy combined with a 24–60 h fast compared to chemotherapy alone [34, 40]. Fasting also has significant benefits in terms of reducing the toxicity of chemotherapy treatment [41].

### Differential stress resistance

During shot-term fasting (STF), normal healthy cells and cancer cells respond differently to chemotherapeutic agents. This phenomenon is termed differential stress resistance (DSR). In normal healthy cells, nutrient deprivation inactivated growth-promoting pathways to prioritize maintenance and repair pathways, leading to increase cellular protection and resistance to multiple stresses. In cancer cells, however, growth-promoting pathways remain over activated and unable to activate the protective response, contributing to less resistance to stress and more vulnerability to chemotherapeutic agents. Because of these differential responses of healthy versus cancer cells to STF, chemotherapy causes more DNA damage and apoptosis in tumor cells, leaving healthy cells unharmed when combined with STF [42, 43].

### Fasting-mimicking diet (FMD)

Considering the potential adverse effects of CR and fasting in some cancer patients, pharmacological agents that mimic CR, such as rapamycin, metformin, and resveratrol, could serve as effective alternatives to achieve the protective effects of CR. In animal models, a fasting-mimicking diet showed a similar reduction in tumor progression as short-term starvation [44]. The administration of the CR mimetic rapamycin (sirolimus), achieved mTOR inhibition, extended lifespan, and delayed cancer in mice [45]. Another CR mimetic, metformin, was shown to suppress tumor development and growth in multiple experimental models, including colon, mammary, and hematopoietic cancer models [46].

Recently, Caffa et al. showed that in mouse models of hormone-receptor-positive breast cancer, periodic fasting or an FMD enhanced the endocrine therapeutics activity of tamoxifen and fulvestrant by lowering circulating IGF1, insulin, and leptin and by inhibiting AKT-mTOR signaling [47]. The authors also showed that in patients with hormone-receptor-positive breast cancer receiving estrogen therapy, cycles of an FMD cause metabolic changes like those observed in mice.

### Alternative dietary approaches

Dietary regimens, such as a low carbohydrate/ketogenic diet, promote ketones derived from fatty acids as energy sources rather than glucose. Such dietary interventions were also shown to reduce tumor growth in several tumor cell models [48–50]. Moreover, there is preclinical and clinical evidence supporting the assumption that ketogenic therapy through fasting and ketogenic diets can enhance radiotherapy [51].

### Clinical data

Clinical data on CR is limited. Nevertheless, data from human trials demonstrate that CR in non-obese individuals results in favorable changes similar to those observed in rodent models [52]. The potential advantage of CR as an adjunct therapy for a range of cancers has recently received significant attention [53].

A small pilot study comprising ten patients diagnosed with breast, prostate, esophageal, or lung cancer in advanced stages suggested that intermittent fasting periods before and after chemotherapy reduce the self-reported side effects, including fatigue and weakness, vomiting, and diarrhea [54]. Another study included 11 patients that examined the feasibility of combining chemotherapy and intermittent fasting during Ramadan’s Muslim fasting month, and it showed that combining fasting and chemotherapy was safe, and the side effects of chemotherapy tended to be less [55].

Dorff et al. reported results from a phase I study (20 patients) with various malignancies treated with platinum-based chemotherapy combined with 24, 48, or 72 h STF to identify the optimal fasting duration [42, 56]. The authors reported that 72 h of short-term fasting was associated with normal lymphocyte counts and maintenance of a normal lymphoid/myeloid ratio, while 24 h STF was not.

In a randomized, cross-over trial compromising 34 patients with breast and ovarian cancer, the study confirmed that compared with the Mediterranean diet, STF for 60 h (from 36 h before chemotherapy to 24 h post-chemotherapy) improved tolerance to chemotherapy, enhanced quality of life, and led to lesser fatigue [57].

In 13 breast cancer patients treated with (neo)-adjuvant chemotherapy, a 48-h starvation period (from 24 h before to 24 h after chemotherapy) was associated with reduced hematological toxicity [58]. A further study that included 17 patients, a special ketogenic diet, the so-called “modified Atkins diet,” reportedly reduces the progression of some advanced cancer patients, especially in individuals who experienced at least 10% loss of their body weight [59]. Likewise, in patients with recurrent glioblastoma, a ketogenic regimen was administered simultaneously with the antiangiogenic drug bevacizumab, induced objective responses in 6 out of 7 patients with a median progression-free survival 20.1 weeks [60].

### The concerns associated with CR

There are several concerns associated with the adoption of CR in cancer patients as cancer patients are at a greater risk of weight loss due to the toxic effect of cancer therapies and cachexia, and sarcopenia from tumor-derived signals to degrade adipose and muscle tissues, to which chronic CR may contribute. However, the impact of a few days of fasting on the bodyweight of humans appears far more modest, and it is largely due to water loss [61]. Another concern is related to the anti-inflammatory effect of CR that could be detrimental to cancer patients who may be immunodeficient due to the disease or its therapy. Moreover, fasting may cause mild side effects, including headaches, dizziness, nausea, dyspepsia, and fatigue.

## Conclusion

As nutrition and metabolism are essential for human physiology, it is not surprising that dietary interventions attract attention as a safe means to limit tumor progression or reinstate disease control by the host immune system. While preclinical studies support the concept that reducing total calorie intake may stimulate anticancer immunity, evidence-driven from earlier clinical studies lack enough power to draw definite conclusions. Multiple trials evaluating these possibilities in patients with distinct types of cancer are ongoing. De Groot et al. [62] and Castejón et al. [63], reported on comprehensive lists of ongoing studies of dietary interventions. Such interventions include CR, intermediate fasting, ketogenic and low carbohydrate diets, and restrictive protein diets for patients with several advanced cancer types. Several trials are designed to investigate the combination of dietary restriction in combination with chemotherapy, radiotherapy, checkpoint inhibitors, or metformin to ameliorate side effects, improve quality of life, or improve outcome. Other trials aimed at assessing the preventive role of CR on age-related chronic diseases, including cancer such as the CALERIE trial.

Dietary intervention trials have inherent challenges to overcome. First, dietary interventions are quite heterogeneous. Second, the studies’ control arms usually receive advice in relation to healthy nutritional habits. Third, compliance with the tested dietary intervention is hard to be enforced and difficult to be monitored. Nevertheless, future research will hopefully inform and firmly establish the potential efficacy and safety of these dietary interventions.

## References

[1] De Pergola G, Silvestris F (2013). Obesity as a major risk factor for cancer. J Obes.

[2] Demark-Wahnefried W, Platz EA, Ligibel JA, Blair CK, Courneya KS, Meyerhardt JA, Ganz PA, Rock CL, Schmitz KH, Wadden T, Philip EJ, Wolfe B, Gapstur SM, Ballard-Barbash R, McTiernan A, Minasian L, Nebeling L, Goodwin PJ (2012). The role of obesity in cancer survival and recurrence. Cancer Epidemiol Biomark Prev.

[3] Lee C, Raffaghello L, Brandhorst S (2012). Fasting cycles retard growth of tumors and sensitize a range of cancer cell types to chemotherapy. Sci Transl Med.

[4] Galluzzi L, Baehrecke EH, Ballabio A (2017). Molecular definitions of autophagy and related processes. EMBO J.

[5] Galluzzi L, Vitale I, Aaronson SA, Abrams JM, Adam D, Agostinis P, Alnemri ES, Altucci L, Amelio I, Andrews DW, Annicchiarico-Petruzzelli M, Antonov AV, Arama E, Baehrecke EH, Barlev NA, Bazan NG, Bernassola F, Bertrand MJM, Bianchi K, Blagosklonny MV, Blomgren K, Borner C, Boya P, Brenner C, Campanella M, Candi E, Carmona-Gutierrez D, Cecconi F, Chan FKM, Chandel NS, Cheng EH, Chipuk JE, Cidlowski JA, Ciechanover A, Cohen GM, Conrad M, Cubillos-Ruiz JR, Czabotar PE, D’Angiolella V, Dawson TM, Dawson VL, de Laurenzi V, de Maria R, Debatin KM, DeBerardinis RJ, Deshmukh M, di Daniele N, di Virgilio F, Dixit VM, Dixon SJ, Duckett CS, Dynlacht BD, el-Deiry WS, Elrod JW, Fimia GM, Fulda S, García-Sáez AJ, Garg AD, Garrido C, Gavathiotis E, Golstein P, Gottlieb E, Green DR, Greene LA, Gronemeyer H, Gross A, Hajnoczky G, Hardwick JM, Harris IS, Hengartner MO, Hetz C, Ichijo H, Jäättelä M, Joseph B, Jost PJ, Juin PP, Kaiser WJ, Karin M, Kaufmann T, Kepp O, Kimchi A, Kitsis RN, Klionsky DJ, Knight RA, Kumar S, Lee SW, Lemasters JJ, Levine B, Linkermann A, Lipton SA, Lockshin RA, López-Otín C, Lowe SW, Luedde T, Lugli E, MacFarlane M, Madeo F, Malewicz M, Malorni W, Manic G, Marine JC, Martin SJ, Martinou JC, Medema JP, Mehlen P, Meier P, Melino S, Miao EA, Molkentin JD, Moll UM, Muñoz-Pinedo C, Nagata S, Nuñez G, Oberst A, Oren M, Overholtzer M, Pagano M, Panaretakis T, Pasparakis M, Penninger JM, Pereira DM, Pervaiz S, Peter ME, Piacentini M, Pinton P, Prehn JHM, Puthalakath H, Rabinovich GA, Rehm M, Rizzuto R, Rodrigues CMP, Rubinsztein DC, Rudel T, Ryan KM, Sayan E, Scorrano L, Shao F, Shi Y, Silke J, Simon HU, Sistigu A, Stockwell BR, Strasser A, Szabadkai G, Tait SWG, Tang D, Tavernarakis N, Thorburn A, Tsujimoto Y, Turk B, vanden Berghe T, Vandenabeele P, Vander Heiden MG, Villunger A, Virgin HW, Vousden KH, Vucic D, Wagner EF, Walczak H, Wallach D, Wang Y, Wells JA, Wood W, Yuan J, Zakeri Z, Zhivotovsky B, Zitvogel L, Melino G, Kroemer G (2018). Molecular mechanisms of cell death: recommendations of the Nomenclature Committee on Cell Death 2018. Cell Death Differ.

[6] Kimmelman AC, White E (2017). Autophagy and tumor metabolism. Cell Metab.

[7] Kroemer G, Mariño G, Levine B (2010). Autophagy and the integrated stress response. Mol Cell.

[8] Galluzzi L, Pietrocola F, Bravo-San Pedro JM (2015). Autophagy in malignant transformation and cancer progression. EMBO J.

[9] Sharifi MN, Mowers EE, Drake LE, Collier C, Chen H, Zamora M, Mui S, Macleod KF (2016). Autophagy promotes focal adhesion disassembly and cell motility of metastatic tumor cells through the direct interaction of Paxillin with LC3. Cell Rep.

[10] Lebovitz CB, Robertson AG, Goya R, Jones SJ, Morin RD, Marra MA, Gorski SM (2015). Cross-cancer profiling of molecular alterations within the human autophagy interaction network. Autophagy.

[11] Mrakovcic M, Fröhlich LF (2018). p53-mediated molecular control of autophagy in tumor cells. Biomolecules.

[12] Chen N, Karantza V (2011). Autophagy as a therapeutic target in cancer. Cancer Biol Ther.

[13] Galluzzi L, Bravo-San Pedro JM, Levine B, Green DR, Kroemer G (2017). Pharmacological modulation of autophagy: therapeutic potential and persisting obstacles. Nat Rev Drug Discov.

[14] Fulda S, Kögel D (2015). Cell death by autophagy: emerging molecular mechanisms and implications for cancer therapy. Oncogene.

[15] Fulda S (2017). Autophagy in cancer therapy. Front Oncol.

[16] Grasso S, Pereira GJS, Palmeira-Dos-Santos C (2016). Autophagy regulates Selumetinib (AZD6244) induced-apoptosis in colorectal cancer cells. Eur J Med Chem.

[17] Palmeira dos Santos C, Pereira GJ, Barbosa CM, Jurkiewicz A, Smaili SS, Bincoletto C (2014). Comparative study of autophagy inhibition by 3MA and CQ on Cytarabine-induced death of leukaemia cells. J Cancer Res Clin Oncol.

[18] Gigli R, Pereira GJ, Antunes F (2016). The biphosphinic paladacycle complex induces melanoma cell death through lysosomal-mitochondrial axis modulation and impaired autophagy. Eur J Med Chem.

[19] Liang B, Kong D, Liu Y, Liang N, He M, Ma S, Liu X (2012). Autophagy inhibition plays the synergetic killing roles with radiation in the multi-drug resistant SKVCR ovarian cancer cells. Radiat Oncol.

[20] Chen Y, Li X, Guo L (2015). Combining radiation with autophagy inhibition enhances suppression of tumor growth and angiogenesis in esophageal cancer. Mol Med Rep.

[21] Vakifahmetoglu-Norberg H, Xia HG, Yuan J (2015). Pharmacologic agents targeting autophagy. J Clin Invest.

[22] Pavlova NN, Thompson CB (2016). The emerging hallmarks of cancer metabolism. Cell Metab.

[23] Beloribi-Djefaflia S, Vasseur S, Guillaumond F (2016). Lipid metabolic reprogramming in cancer cells. Oncogenesis.

[24] Renehan AG, Egger M, Minder C, O'Dwyer ST, Shalet SM, Zwahlen M (2005). IGF-I, IGF binding protein-3 and breast cancer risk: comparison of 3 meta-analyses. Int J Cancer.

[25] Renehan AG, Zwahlen M, Minder C, O'Dwyer ST, Shalet SM, Egger M (2004). Insulin-like growth factor (IGF)-I, IGF binding protein-3, and cancer risk: systematic review and meta-regression analysis. Lancet.

[26] Di Francesco A, Di Germanio C, Bernier M, de Cabo R (2018). A time to fast. Science.

[27] Speakman JR, Mitchell SE (2011). Caloric restriction. Mol Asp Med.

[28] Hursting SD, Dunlap SM, Ford NA, Hursting MJ, Lashinger LM (2013). Calorie restriction and cancer prevention: a mechanistic perspective. Cancer Metab.

[29] Longo VD, Mattson MP (2013). Fasting: molecular mechanisms and clinical applications. Cell Metab.

[30] Imayama I, Ulrich CM, Alfano CM, Wang C, Xiao L, Wener MH, Campbell KL, Duggan C, Foster-Schubert KE, Kong A, Mason CE, Wang CY, Blackburn GL, Bain CE, Thompson HJ, McTiernan A (2012). Effects of a caloric restriction weight loss diet and exercise on inflammatory biomarkers in overweight/obese postmenopausal women: a randomized controlled trial. Cancer Res.

[31] Berrigan D, Lavigne JA, Perkins SN, Nagy TR, Barrett JC, Hursting SD (2005). Phenotypic effects of calorie restriction and insulin-like growth factor-1 treatment on body composition and bone mineral density of C57BL/6 mice: implications for cancer prevention. In Vivo.

[32] Bowers LW, Rossi EL, O'Flanagan CH, deGraffenried LA, Hursting SD (2015). The role of the insulin/IGF system in cancer: lessons learned from clinical trials and the energy balance-cancer link. Front Endocrinol (Lausanne).

[33] Mukherjee P, Abate LE, Seyfried TN (2004). Antiangiogenic and proapoptotic effects of dietary restriction on experimental mouse and human brain tumors. Clin Cancer Res.

[34] Bianchi G, Martella R, Ravera S (2015). Fasting induces anti-Warburg effect that increases respiration but reduces ATP-synthesis to promote apoptosis in colon cancer models. Oncotarget.

[35] Mattison JA, Colman RJ, Beasley TM, Allison DB, Kemnitz JW, Roth GS, Ingram DK, Weindruch R, de Cabo R, Anderson RM (2017). Caloric restriction improves health and survival of rhesus monkeys. Nat Commun.

[36] Lo Re O, Panebianco C, Porto S, Cervi C, Rappa F, di Biase S, Caraglia M, Pazienza V, Vinciguerra M (2018). Fasting inhibits hepatic stellate cells activation and potentiates anti-cancer activity of Sorafenib in hepatocellular cancer cells. J Cell Physiol.

[37] Caffa I, D'Agostino V, Damonte P (2015). Fasting potentiates the anticancer activity of tyrosine kinase inhibitors by strengthening MAPK signaling inhibition. Oncotarget.

[38] Caccialanza R, Cereda E, De Lorenzo F, Farina G, Pedrazzoli P, AIOM-SINPE-FAVO Working Group (2018). To fast, or not to fast before chemotherapy, that is the question. BMC Cancer.

[39] Sancar A, Lindsey-Boltz LA, Gaddameedhi S, Selby CP, Ye R, Chiou YY, Kemp MG, Hu J, Lee JH, Ozturk N (2015). Circadian clock, cancer, and chemotherapy. Biochemistry.

[40] Safdie F, Brandhorst S, Wei M, Wang W, Lee C, Hwang S, Conti PS, Chen TC, Longo VD (2012). Fasting enhances the response of glioma to chemo- and radiotherapy. PLoS One.

[41] Raffaghello L, Lee C, Safdie FM, Wei M, Madia F, Bianchi G, Longo VD (2008). Starvation-dependent differential stress resistance protects normal but not cancer cells against high-dose chemotherapy. Proc Natl Acad Sci U S A.

[42] Cheng CW, Adams GB, Perin L, Wei M, Zhou X, Lam BS, da Sacco S, Mirisola M, Quinn DI, Dorff TB, Kopchick JJ, Longo VD (2014). Prolonged fasting reduces IGF-1/PKA to promote hematopoietic-stem-cell-based regeneration and reverse immunosuppression. Cell Stem Cell.

[43] Brandhorst S, Choi IY, Wei M, Cheng CW, Sedrakyan S, Navarrete G, Dubeau L, Yap LP, Park R, Vinciguerra M, di Biase S, Mirzaei H, Mirisola MG, Childress P, Ji L, Groshen S, Penna F, Odetti P, Perin L, Conti PS, Ikeno Y, Kennedy BK, Cohen P, Morgan TE, Dorff TB, Longo VD (2015). A periodic diet that mimics fasting promotes multi-system regeneration, enhanced cognitive performance, and healthspan. Cell Metab.

[44] Pietrocola F, Pol J, Vacchelli E (2016). Caloric restriction mimetics enhance anticancer immunosurveillance. Cancer Cell.

[45] Harrison DE, Strong R, Sharp ZD, Nelson JF, Astle CM, Flurkey K, Nadon NL, Wilkinson JE, Frenkel K, Carter CS, Pahor M, Javors MA, Fernandez E, Miller RA (2009). Rapamycin fed late in life extends lifespan in genetically heterogeneous mice. Nature.

[46] Pollak MN (2012). Investigating metformin for cancer prevention and treatment: the end of the beginning. Cancer Discov.

[47] Caffa I, Spagnolo V, Vernieri C, Valdemarin F, Becherini P, Wei M, Brandhorst S, Zucal C, Driehuis E, Ferrando L, Piacente F, Tagliafico A, Cilli M, Mastracci L, Vellone VG, Piazza S, Cremonini AL, Gradaschi R, Mantero C, Passalacqua M, Ballestrero A, Zoppoli G, Cea M, Arrighi A, Odetti P, Monacelli F, Salvadori G, Cortellino S, Clevers H, de Braud F, Sukkar SG, Provenzani A, Longo VD, Nencioni A (2020). Fasting-mimicking diet and hormone therapy induce breast cancer regression. Nature.

[48] Ho VW, Leung K, Hsu A, Luk B, Lai J, Shen SY, Minchinton AI, Waterhouse D, Bally MB, Lin W, Nelson BH, Sly LM, Krystal G (2011). A low carbohydrate, high protein diet slows tumor growth and prevents cancer initiation. Cancer Res.

[49] Caso J, Masko EM, Ii JA (2013). The effect of carbohydrate restriction on prostate cancer tumor growth in a castrate mouse xenograft model. Prostate.

[50] Stafford P, Abdelwahab MG, Kim DY, Preul MC, Rho JM, Scheck AC (2010). The ketogenic diet reverses gene expression patterns and reduces reactive oxygen species levels when used as an adjuvant therapy for glioma. Nutr Metab (Lond).

[51] Klement RJ (2018). Fasting, fats, and physics: combining ketogenic and radiation therapy against cancer. Complement Med Res.

[52] Most J, Tosti V, Redman LM, Fontana L (2016). Calorie restriction in humans: an update. Ageing Res Rev.

[53] Saleh AD, Simone BA, Palazzo J, Savage JE, Sano Y, Dan T, Jin L, Champ C, Zhao S, Lim M, Sotgia F, Camphausen K, Pestell R, Mitchell J, Lisanti M, Simone NL (2013). Caloric restriction augments radiation efficacy in breast cancer. Cell Cycle.

[54] Safdie FM, Dorff T, Quinn D (2009). Fasting and cancer treatment in humans: a case series report. Aging (Albany NY).

[55] Badar T, Ismail A, Alshanqqeeti A (2014) Safety and feasability of Muslim fasting while receiving chemotherapy. IOSR J Pharm 4:15–20. 10.9790/3013-0401015-20

[56] Dorff TB, Groshen S, Garcia A, Shah M, Tsao-Wei D, Pham H, Cheng CW, Brandhorst S, Cohen P, Wei M, Longo V, Quinn DI (2016). Safety and feasibility of fasting in combination with platinum-based chemotherapy. BMC Cancer.

[57] Bauersfeld SP, Kessler CS, Wischnewsky M, Jaensch A, Steckhan N, Stange R, Kunz B, Brückner B, Sehouli J, Michalsen A (2018). The effects of short-term fasting on quality of life and tolerance to chemotherapy in patients with breast and ovarian cancer: a randomized cross-over pilot study. BMC Cancer.

[58] de Groot S, Vreeswijk MP, Welters MJ (2015). The effects of short-term fasting on tolerance to (neo) adjuvant chemotherapy in HER2-negative breast cancer patients: a randomized pilot study. BMC Cancer.

[59] Tan-Shalaby JL, Carrick J, Edinger K, Genovese D, Liman AD, Passero VA, Shah RB (2016). Modified Atkins diet in advanced malignancies - final results of a safety and feasibility trial within the Veterans Affairs Pittsburgh Healthcare System. Nutr Metab (Lond).

[60] Rieger J, Bähr O, Maurer GD (2014). ERGO: a pilot study of ketogenic diet in recurrent glioblastoma. Int J Oncol.

[61] Kerndt PR, Naughton JL, Driscoll CE, Loxterkamp DA (1982). Fasting: the history, pathophysiology and complications. West J Med.

[62] de Groot S, Pijl H, van der Hoeven JJM, Kroep JR (2019). Effects of short-term fasting on cancer treatment. J Exp Clin Cancer Res.

[63] Castejón M, Plaza A, Martinez-Romero J, Fernandez-Marcos PJ, Cabo R, Diaz-Ruiz A (2020). Energy restriction and colorectal cancer: a call for additional research. Nutrients.

